# *Enterococcus faecalis* Infection and Reactive Oxygen Species Down-Regulates the miR-17-92 Cluster in Gastric Adenocarcinoma Cell Culture

**DOI:** 10.3390/genes5030726

**Published:** 2014-08-28

**Authors:** Jesper A. B. Strickertsson, Lene Juel Rasmussen, Lennart Friis-Hansen

**Affiliations:** 1 Center for Healthy Aging, Department of Cellular and Molecular Medicine, University of Copenhagen, Copenhagen 2200, Denmark; E-Mails: jabs@sund.ku.dk (J.A.B.S.); lenera@sund.ku.dk (L.J.R.); 2 Center for Genomic Medicine, Rigshospitalet, University of Copenhagen, Copenhagen 2100, Denmark; 3 Department of Clinical Biochemistry, Næstved Hospital, Næstved 4700, Denmark

**Keywords:** miRNA, reactive oxygen species, *E. faecalis*, miR-17-92 cluster, infection

## Abstract

Chronic inflammation due to bacterial overgrowth of the stomach predisposes to the development of gastric cancer and is also associated with high levels of reactive oxygen species (ROS). In recent years increasing attention has been drawn to microRNAs (miRNAs) due to their role in the pathogenesis of many human diseases including gastric cancer. Here we studied the impact of infection by the gram-positive bacteria *Enterococcus faecalis* (*E. faecalis*) on global miRNA expression as well as the effect of ROS on selected miRNAs. Human gastric adenocarcinoma cell line MKN74 was infected with living *E. faecalis* for 24 h or for 5 days or with *E. faecalis* lysate for 5 days. The miRNA expression was examined by microarray analysis using Affymetrix GeneChip miRNA Arrays. To test the effect of ROS, MKN74 cells were treated with 100 mM tert-Butyl hydroperoxide (TBHP). Following 5 days of *E. faecalis* infection we found 91 differentially expressed miRNAs in response to living bacteria and 2 miRNAs responded to *E. faecalis* lysate. We verified the down-regulation of the miR-17-92 and miR-106-363 clusters and of other miRNAs involved in the oxidative stress-response by qRT-PCR. We conclude that only infection by living *E. faecalis* bacteria caused a significant global response in miRNA expression in the MKN74 cell culture. *E. faecalis* infection as well as ROS stimulation down-regulated the expression of the miR-17-92 cluster. We believe that these changes could reflect a general response of gastric epithelial cells to bacterial infections.

## 1. Introduction

Globally gastric cancer is the second most common cause of cancer related death [1,2]. Gastric cancer usually develops over decades with bacterial overgrowth and chronic inflammation being some of the main causes of cancer development. Chronic inflammation is not a significant cause of gastric cancer but is believed to be involved in the pathogenesis of about 25% of all cancer cases worldwide [3]. Chronic infection by *Helicobacter pylori* (*H. pylori*) is one of the most common risk factors for developing gastric cancer [4], and can affect the gastric pH balance causing achlorhydria, increasing the risk of gastric cancer development [5]. The increased cancer risk in achlorhydric individuals could be due to bacterial overgrowth of other bacteria than *H. pylori* in the gastric lumen [6]. We have previously shown that the predominant bacteria found in stomachs of achlorhydric mice are gram-positive *Enterococci* species, which are able to survive at low pH [7,8].

*Enterococcus faecalis* (*E. faecalis*) is a gram-positive bacterium capable of inducing genetic instability and mitochondrial dysfunction in epithelial cells through down-regulation of essential DNA damage repair pathways and induction of oxidative DNA damage [9,10,11]. Reactive oxygen species (ROS) consists of free radicals including hydrogen peroxide, superoxide, and hydroxyl radicals and function as intracellular and intercellular signaling molecules. However imbalances in the delicate balance of ROS levels can induce malignant changes in cells through DNA damage and alterations in cellular signaling [12,13]. The intracellular level of ROS is often elevated in cancer cells and high levels of ROS can induce DNA damage, chromosomal instability, DNA double strand breaks and alter cellular signaling pathways [13,14,15]. Conversely, alterations in cellular signaling pathways induced by ROS can have anti-carcinogenic effects on the cells, by reducing tumor growth and angiogenesis through inhibition of cyclin D1 and vascular endothelial growth factor (VEGF) and by inducing apoptosis through, e.g., inhibition of survivin [16,17,18]. One mechanisms behind the anti-carcinogenic effect elicited by ROS is through inhibition of oncogenic microRNA (miRNA) expression [17,18]. In recent years increasing attention has been drawn to miRNAs due to their role in the pathogenesis of many human diseases including gastric cancer [19,20,21,22,23]. miRNAs are short non-coding single-stranded RNA molecules of approximately 19–25 nucleotides which function at the posttranscriptional level as negative regulators of gene expression [24,25]. Several miRNAs are involved in response to DNA damage and in the regulation of ROS. For tables on regulatory miRNAs in the DNA damage response and ROS response see Wan *et al.* [26]. The miRNAs can furthermore function as either tumor suppressors or oncogenes, thereby regulating carcinogenesis [19,21,27,28]. The miR-17-92 cluster is an example of miRNAs with both oncogenic and tumor suppressive properties. It is a polycistronic miRNA consisting of six miRNAs involved in cell proliferation, differentiation, survival, and angiogenesis [29]. The individual miRNAs of this cluster are found in high numbers in tissue of different cancer types and are among the highest expressed miRNAs in gastric cancer [22,30,31]. miRNAs of this cluster are found to be down-regulated during DNA damage via a p53-depedent mechanism or during treatment with ROS [17,26].

Inflamed tissue is normally infiltrated with immune cells making it difficult to dissect the action of the immune cells from that of the lining mucosal cells. Using an *in vitro* tissue culture model allowing us to examine how isolated mucosal cells respond to bacteria, we previously demonstrated that infection by *E. faecalis* caused an inflammatory response, and induced ROS production and DNA damage in MKN74 cells [11]. To further investigate how *E. faecalis* affects the gastric adenocarcinoma cell culture, we examined the effect of bacterial overgrowth by *E. faecalis* on miRNA expression with focus on the miR-17-92 cluster.

## 2. Experimental

### 2.1. Cell Culture, E. faecalis, and Growth Conditions

Human MKN74 gastric adenocarcinoma cell cultures were grown in RPMI 1640 medium (Life Technologies, Carlsbad, CA, USA), supplemented with 10% fetal bovine serum (FBS) (Life Technologies), 100 μg/mL streptomycin, and 100 U/mL penicillin (Life Technologies) at 37 °C, under 5% CO_2_ humidified atmosphere. Infections were performed with *E. faecalis* strain ATCC 29212, grown on 5% blood agar plates at 37 °C. Optical density (OD) of bacteria resuspended in PBS was measured at 550 nm on a UV-1601 spectrophotometer (Shimadzu, Kyoto, Japan). *E. faecalis* lysate was prepared by freeze/thawing the bacterial suspension three times, while sonicating the suspension between each cycle.

### 2.2. Infection of Gastric Cells

MKN74 cells were infected in triplicates with *E. faecalis* for 24 h or 5 days. For 24 h infections, 80% confluent MKN74 cells were washed three times in PBS and incubated in antibiotic free medium. Overnight-grown colonies of *E. faecalis* were resuspended in PBS and added to the MKN74 cell culture at a multiplicity of infection (MOI) of 50 bacteria per cell. 5 day infections were carried out by treating 65% confluent MKN74 cells with living *E. faecalis* at a MOI of 10 or with a lysate protein concentration of 40 μg/μL. Every 24 h, cells were washed three times with PBS and fresh medium and bacteria or lysate was added. Control cells were processed similarly in the absence of bacteria or bacterial lysate.

### 2.3. Tert-Butyl Hydroperoxide Stimulation

Twenty-four hours prior to stimulation, 4 × 10^5^ MKN74 cells were plated in 6-well plates. Cells were treated with 100 mM tert-Butyl hydroperoxide (TBHP) (Sigma-Aldrich, St. Louis, MO, USA) in RPMI1640 growth media (Life Technologies) for 20 min, 40 min, 1 h, 3 h, and 5 h. Treatments were stopped by removing the media and adding 1 mL Trizol reagent (Life Technologies) to each well.

### 2.4. RNA Isolation

RNA was extracted by adding Trizol reagent (Life Technologies) to each culture flask. RNA was isolated according to the manufactures protocol. RNA concentrations were measured on a NanoDrop ND-1000 Spectrophotometer. RNA integrity numbers (RIN) were measured on a 2100 Bioanalyzer (Agilent Technologies, Santa Clara, CA, USA) according to the manufactures protocol.

### 2.5. Microarray 

RNA with a RIN value of 8 or above from three controls and three infected samples (with living *E. faecalis* or with *E. faecalis* lysate 40 μg/μL) for 5 day experiments were submitted to the RH Microarray Center at Copenhagen University Hospital. RNA was amplified and labeled using the 3' IVT Express kit (Affymetrix, Santa Clara, CA, USA) according to manufactures instructions. 250 ng total RNA was used as input. The labeled samples were hybridized to the Affymetrix GeneChip miRNA Array (Affymetrix, Santa Clara, CA, USA). The arrays were washed and stained with phycoerytrin conjugated streptavidin (SAPE) using the Affymetrix Fluidics Station^®^ 450 (Affymetrix), and the arrays were scanned in the Affymetrix GeneArray^®^ 2500 scanner (Affymetrix) to generate fluorescent images, as described in the Affymetrix GeneChip^®^ protocol. 12 raw cell intensity files (CEL files) were generated in the GeneChip^®^ Command Console^®^ Software 3.0 (AGCC) [32]. The raw CEL-files were made publicly available at ArrayExpress with accession number E-MEXP-3694. We defined miRNAs with a difference in arbitrary expression units ≥20 when compared to controls, and a *p*-value <0.05 as being differentially expressed.

### 2.6. Quantitative Reverse Transcription PCR

MicroRNA expression was measured and quantified using TaqMan^®^ MicroRNA Assays for each miRNA, according to the manufacturer’s protocol (Applied Biosystems, Foster City, CA, USA). For each of the three biological samples technical triplicates were made. Analyses were carried out on the ABI Prism^®^ 7900HT Sequence Detection System (SDS) (Applied Biosystems). For *E. faecalis* treated cells miR-17-92 cluster miRs were normalized to hsa-miR-191, and for TBHP treated cells the miR-17-92 cluster miRs were normalized to RNU48 (Applied Biosystems). miR-27a*, miR-27b*, and miR-24-2* measured in cells treated with *E. faecalis* or TBHP were normalized to RNU44 (Applied Biosystems). These served as endogenous controls, and relative expression was calculated using the 2^−ΔΔCt^ method [33].

### 2.7. Statistical Analysis

Quantitative reverse transcription PCR (qRT-PCR) results were expressed as mean values of at least three independent experiments measured in three technical replicates, and analyzed by unpaired two-tailed *t*-test. The differences between data sets were considered significant at *p*-values ≤0.05. Error bars = ±SEM unless otherwise indicated.

## 3. Results and Discussion

### 3.1. Living E. faecalis Affect Global miRNA Expression

We first examined the global changes in miRNA expression following infection with living *E. faecalis* and with *E. faecalis* lysate. To evaluate the global changes in miRNA expression we used principal component analysis (PCA) of the microarrays, which showed that the MKN74 cells infected with living *E. faecalis* markedly changed the miRNA transcription profile compared to that of the corresponding uninfected control cells (Supplementary Figure S1). In contrast MKN74 cells treated with *E. faecalis* lysate grouped together with the corresponding uninfected MKN74 cells indicating that the lysate did not affect miRNA transcription in the cell culture (Supplementary Figure S1).

### 3.2. E. faecalis Infection Alters the Expression of the miR-17-92 Cluster, miR-106-363 Cluster, and Other ROS Responsive miRNAs

**Figure 1:**
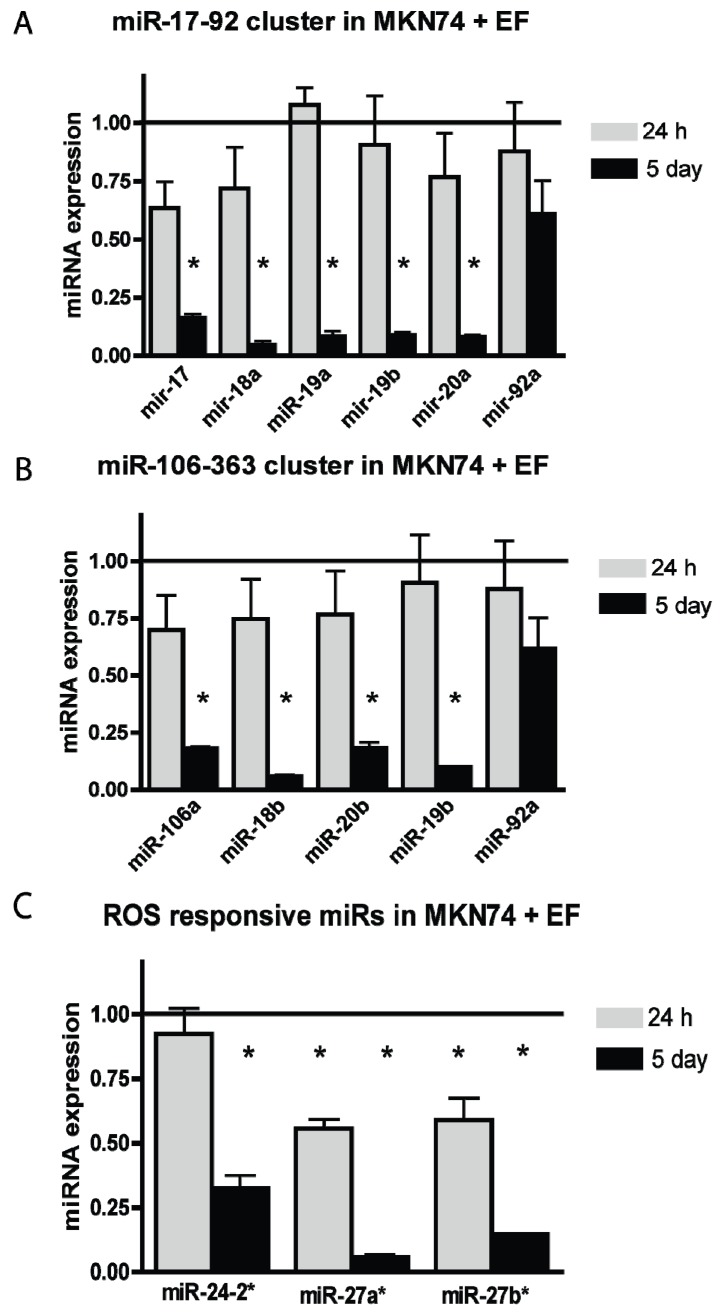
miR-17-92, miR-106-363, and ROS responsive miRs were down-regulated in MKN74 cell culture after 24 h or 5 days of infection with *E. faecalis*. (**A**) qRT-PCR showed that 24 h of infection caused a down-regulation in the expression of miRNAs in the miR-17-92 cluster as well as a significant down-regulation of the cluster following 5 days of infection; (**B**) A similar pattern was observed for the paralogous miR-106-363 cluster following *E. faecalis* infection; (**C**) 24 h of infection by *E. faecalis* caused a significant down-regulation in the expression of ROS responsive miRNAs miR-27a* and miR-27b*. After 5 days of infection miR-24-2* was also significantly down-regulated. Expression of uninfected control cells = 1. Each group consisted of three biological samples run in three technical triplicates. * denotes significantly different from untreated cells *p* < 0.05.

Following microarray analysis we found 91 miRNAs that were differentially expressed in response to living bacteria and only 2 miRNAs responded to treatment with bacterial lysate (Supplementary Tables S1 and S2). Among the differentially expressed miRNAs we found miRNAs of the miR-17-92 cluster, the miR-106-363 cluster and miRNAs involved in oxidative stress-response (Table 1). The changes in miRNA expression in infected MKN74 cell culture were validated using qRT-PCR (Figure 1). First we examined the miRNAs of the miR-17-92 cluster. After 24 h of infection miR-17, miR-18a, and miR-20a were down-regulated; however, this trend was not significant. After 5 days of infection all miRNAs in the miR-17-92 cluster were significantly down-regulated as compared to uninfected cells with the exception of miR-92 (Figure 1A). A similar pattern was observed for the paralogous miR-106-363 cluster showing a non-significant down-regulation in expression of miR-106a, miR-18a, and miR-20b after 24 h and a highly significant down-regulation of miR-106a, miR-18a, miR-20b, and miR-19b after 5 days of *E. faecalis* infection (Figure 1B). We have previously shown that *E. faecalis* induce ROS in tissue culture. Therefore, we next examined the expression of three ROS responsive miRNAs, which previously have been shown to be down-regulated in response to ROS stimulation [17,34]. miR-27a* and miR-27b* was approximately 50% reduced after 24 h of infection and after 5 days miR-24-2*, miR-27a*, and miR-27b* were down-regulated by 75%–90% (Figure 1C). Our new findings are consistent with the presence of ROS both after 24 h and 5 days of infection with living bacteria.

### 3.3. TBHP Reduce the Expression of miRNAs from the miR-17-92 Cluster in a Time Dependent Manner

**Figure 2:**
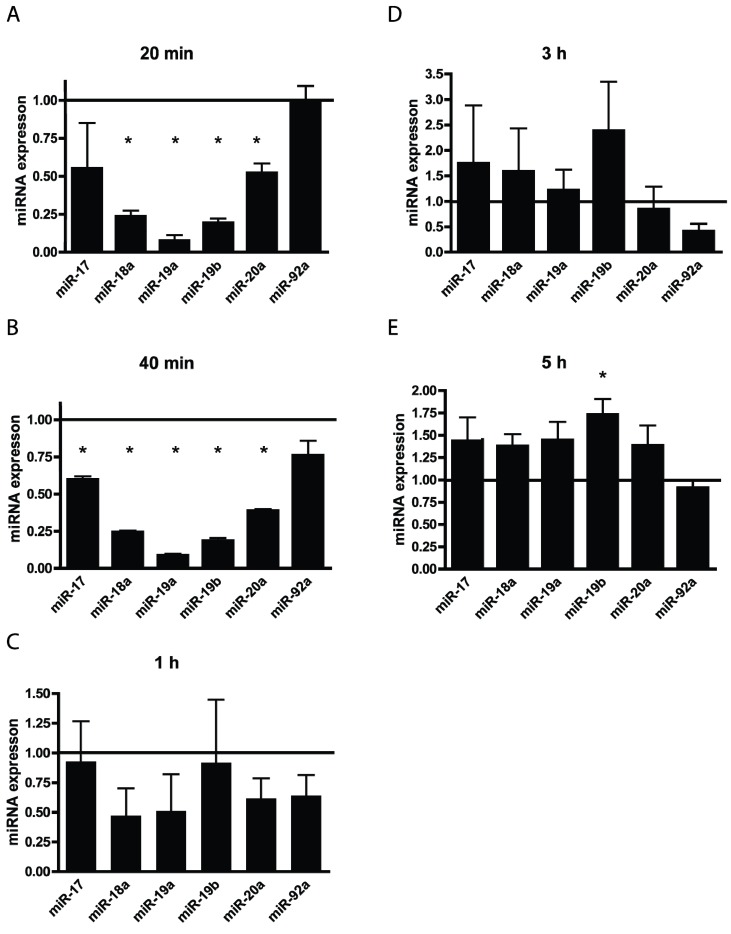
Tert-Butyl hydroperoxide (TBHP) reduces the expression of miRNAs from the miR-17-92 cluster in a time dependent manner. (**A**) After 20 min of TBHP treatment miR-18a, miR-19a, miR-19b, and miR-20a were significantly down-regulated; (**B**) At 40 min miR-17 was also down-regulated; (**C**,**D**) 1 h and 3 h of treatment did not alter the expression of the miR-17-92 cluster significantly. (**E**) After 5 h the miRNAs of the miR-17-92 cluster was slightly up-regulate; however, this trend was only significant for miR-19b. Expression of uninfected control cells = 1. Each group consisted of three biological samples run in three technical triplicates. * denotes significantly different from untreated cells *p* < 0.05.

We have previously shown that *E. faecalis* infection causes an increase in ROS production independently of oxidative phosphorylation [11]. Having seen a changed expression of ROS responsive miRNAs we wanted to examine if the changes in the expression of all the individual miRNAs of the miR-17-92 cluster could be induced by ROS stimulation. We treated the MKN74 cells exogenously with TBHP (TBHP mimics ROS activity), letting us examine the acute response of cultured cells to a high load of ROS, as opposed to long-term stimulation with low dose, simulating chronic inflammation. We observed that treatment with TBHP impaired miRNAs from the miR-17-92 cluster (Figure 2). After 20 min miR-18a, miR-19a, miR-19b, and miR-20a were significantly down-regulated (Figure 2A). At 40 min of ROS induction miR-17 was also down-regulated thus giving a similar response as 5 days of *E. faecalis* infection (Figure 1A and Figure 2B). In contrast, ROS stimulation of 1, 3, and 5 h did not significantly alter expression of the miR-17-92 cluster, with the exception of miR-19b, which was up-regulated after 5 h (Figure 2C–E). ROS are highly reactive and therefore short lived in cell culture media, which can explain why no effect on the ROS responsive miRNAs was observed during long incubation times (Figure 2C–E). Thus the results of the short incubation times (Figure 2A,B), as opposed to the longer incubation times (Figure 2C–E) were comparable to our results during long-term infection (Figure 1A).

**Table 1:** Table showing microarray fold change data of selected miRNAs from MKN74 cells treated with living *E. faecalis* for 5 days compared to uninfected control cells. Only the miRNAs validated by quantitative reverse transcription PCR (qRT-PCR) are shown. Each group consisted of three biological replicates.

miRNA	Fold Change	Difference of Arbitrary Expression	*p*-Value	Corresponding miRNA Cluster
hsa-miR-17_st	−1.8	630.9	0.0001	miR-17-92 cluster
hsa-miR-18a_st	−9.9	245.7	>0.0001	
hsa-miR-20a_st	−4.5	526.9	>0.0001	
hsa-miR-19b_st	−4.1	271.2	0.0002	miR-17-92 and miR-106-363 cluster
hsa-miR-92a_st	1.4	−563.5	0.0023	
hsa-miR-18b_st	−15.8	33.4	0.0001	miR-106-363 cluster
hsa-miR-20b_st	−8.3	92.2	>0.0001	
hsa-miR-106a_st	−1.9	580.9	>0.0001	
hsa-miR-24-2-star_st	−3.6	53.4	0.0011	ROS responsive miRs
hsa-miR-27a-star_st	−14.7	54.0	0.0001	

Among the 91 miRNAs regulated during live *E. faecalis* infection, we observed down-regulation of several miRNAs previously shown to be down-regulated in response to ROS. Among the differentially expressed miRNAs we found six miRNAs from the miR-17-92 cluster and five miRNAs from the paralogous miR-106-363 cluster. These were significantly down-regulated after five days of infection and after ROS treatment, supporting our previous findings of elevated intracellular ROS production during 5 days of *E. faecalis* infection. Other studies have also found a down-regulation of miRNAs from these clusters in response to ROS or ROS inducing agents [17,18]. The miR-17-92 cluster has been found to inhibit the generation of ROS and DNA damage [35]. Thus it is tempting to speculate that the inhibition of miR-17-92 by ROS creates a negative feedback loop resulting in even more ROS and DNA damage. DNA damage affect the miR-17-92 cluster causing its down-regulation by the DNA damage responsive tumor protein p53 [36] thus the mechanism behind ROS mediated miR-17-92 down-regulation could be through DNA damage induced by ROS, and it has been suggested that down-regulated miR-17-92 could serve as a marker for DNA damage or ROS [35,37]. Former infection studies using the carcinogenic bacteria *H. pylori* show that bacterial induced ROS in the host cells increase tumorigenisity through increased DNA damage and genomic instability [38,39]. However, based on this study it is not possible to conclude if bacterial induced down-regulation of the discussed miRNAs and induction of ROS has a tumor suppressive or a tumor promoting effect on the cells. Known mechanisms behind ROS and miRNA mediated anti-tumor effects include induction of apoptosis and reduction of growth signals, and these parameters still need to be addressed in future studies of *E. faecalis* infected gastric cancer cells [17,40].

The miR-17-92 cluster is widely studied and has been characterized with both oncogenic and tumor suppressive properties [29]. Overexpression of miR-17-92 has been suggested to contribute to tumor formation by reducing DNA damage and ROS to a level that does not kill the cell whilst inhibiting repressors of proliferation leading to survival of damaged cells [30,35]. Some of the individual miRNAs of the cluster have furthermore been shown to have oncogenic properties. miR-18a is found in high levels in gastric adenocarcinoma tissue where it targets STAT3 inhibitors leading to downstream activation of the cell-proliferation gene c-Myc, and antiapoptotic genes such as Bcl-xL and Survivin [41]. Expression of the miRNAs miR-19a and miR-19b is also found up-regulated in gastric cancer tissue where they contribute to multi drug resistance in the host cell by targeting the important tumor suppressor protein PTEN [42]. miR-92a is among the most consistently up-regulated miRNAs in gastric cancer [31], and miR-20a is involved in the carcinogenesis of gastric cancer through modulation of the EGR2 signaling pathway [43]. Because the miRNAs of this cluster are associated with pro carcinogenic effects, bacterial induced inhibition of miR-17-92 expression could protect the host from tumor development. On the other hand, the miR-17-92 cluster can also have tumor suppressive properties by inhibiting c-MYC induced cell proliferation through down-regulation of E2Fs which drive cell cycle progression from G_1_ into S phase [29]. A loss of the cluster as we observe in this study could therefore promote cell proliferation and carcinogenesis. Furthermore, miR-17 and miR-20a have been reported to have anti-carcinogenic effects by negatively regulating oncogenic cyclin D1 [44]. Chronic bacterial infection exposes cells to cytotoxic levels of ROS and constant growth signal stimulation, thus a loss of miR-17-92 expression during infection could promote cell proliferation and in this way contribute to tumor development. Interestingly miR-92 was the only miRNA in this study not affected by either *E. faecalis* infection or ROS treatment. This miRNA induce proliferation, migration, and invasion by targeting PTEN in colorectal cells [45]. As individual miRNAs may target several hundred mRNAs involved in different pathways, the outcome of miR-17-92 down-regulation is likely to be dependent on the pattern and level of regulation of the individual miRNAs and also on cell type and environmental factors.

## 4. Conclusions

In conclusion, our results demonstrate that infection by living *E. faecalis*, but not lysate, causes a reduction in miR-17-92 and miR-106-363 cluster miRNAs as well as miR-24-2 and miR-27a/b, and that ROS significantly reduce the expression of miR-17-92. Based on our previous results showing that *E. faecalis* infection induces ROS in the host cells and on miRNA expression data presented in this study and by other groups we propose that down-regulation of miRNAs of the miR-17-92 cluster, miR-106-363 cluster and miR-24-2 and miR-27a/b could be a consequence of ROS induced DNA damage by the bacterial infection. However this claim needs to be further investigated.

As several pathogenic bacteria have been shown to affect numerous parameters in gastric host cells such as ROS production, DNA damage response and host genome integrity, the changes of miRNA expression observed in this study could reflect a general reaction to bacterial infection and may not be unique for *E. faecalis* [11,38,39,46]. Further studies investigating the effect on gastric epithelial miRNA expression of other pathogenic bacteria known to have detrimental effects on human health such as *H. pylori* or *Escherichia coli* should be performed as well as studies using non-pathogenic bacteria. Understanding the common molecular mechanisms underlying gastric infection and carcinogenesis is important for early identification or identifying individuals at high risk of gastric cancer, and for rising strategies to combat this malignancy.

## Supplementary Files

Supplementary File 1
