# Supplementary material for: Enterococcus faecalis Infection and Reactive Oxygen Species Down-Regulates the miR-17-92 Cluster in Gastric Adenocarcinoma Cell Culture
Source: Genes (Basel). 2014 Aug 28;5(3):726–38. doi: 10.3390/genes5030726 (PMC4198927; doi:10.3390/genes5030726)
Supplement: Supplementary File 1 [file genes-05-00726-s001.docx]

Supplementary Information

**Figure S1.** PCA plot showing differentially expressed miRNAs for the two treatments. The miRNA transcription profile of cells infected for 5 days with living *E. faecalis* (red) was significantly different compared to the corresponding uninfected control cells (green). The transcription profile of cells infected with *E. faecalis* lysate and that of the corresponding uninfected MKN74 cells grouped together indicating that the lysate did not affect miRNA expression in the MKN74 cell culture (blue and purple).

**Table S1.** Microarray data from MKN74 cells treated with viable *E. faecalis* showing the differentially expressed miRNAs filtered by difference of arbitrary expression ≥20 and
*p*-value ≤0.05.

| miRNA | Difference of Arbitrary Expression | Fold Change | *p*-value |
| --- | --- | --- | --- |
| hsa-miR-18b_st | 33.4 | −15.8 | 0.0001 |
| hsa-miR-27a-star_st | 54.0 | −14.7 | 0.0001 |
| hsa-miR-503_st | 39.9 | −11.6 | 0.0006 |
| hsa-miR-18a_st | 245.7 | −9.9 | >0.0001 |
| hsa-miR-20b_st | 92.2 | −8.3 | >0.0001 |
| hsa-miR-106b_st | 315.1 | −8.1 | >0.0001 |
| hsa-miR-15a_st | 21.6 | −7.5 | 0.0010 |
| hsa-miR-200a_st | 24.7 | −7.4 | 0.0001 |
| hsa-miR-21-star_st | 46.4 | −7.3 | 0.0028 |
| hsa-miR-34a_st | 85.5 | −6.3 | 0.0002 |
| hsa-miR-194_st | 30.6 | −6.2 | 0.0011 |
| hsa-miR-584_st | 86.1 | −6.2 | 0.0001 |
| hsa-miR-29b-1-star_st | 102.3 | −5.1 | 0.0070 |
| hsa-miR-126_st | 20.4 | −4.9 | >0.0001 |
| hsa-miR-141_st | 21.7 | −4.8 | >0.0001 |
| hsa-miR-27b_st | 168.6 | −4.6 | >0.0001 |
| hsa-miR-934_st | 62.3 | −4.6 | 0.0112 |
| hsa-miR-20a_st | 526.9 | −4.5 | >0.0001 |
| hsa-miR-181d_st | 27.5 | −4.2 | 0.0054 |
| hsa-miR-19b_st | 271.2 | −4.1 | 0.0002 |
| hsa-miR-21_st | 120.0 | −4.0 | >0.0001 |
| hsa-miR-7_st | 42.7 | −3.8 | 0.0001 |
| hsa-miR-24-2-star_st | 53.4 | −3.6 | 0.0011 |
| hsa-miR-27a_st | 753.1 | −3.4 | >0.0001 |
| hsa-let-7g_st | 64.3 | −3.4 | 0.0004 |
| hsa-miR-421_st | 69.5 | −3.3 | 0.0002 |
| hsa-miR-210_st | 118.8 | −3.2 | 0.0009 |
| hsa-miR-324-5p_st | 75.9 | −3.1 | >0.0001 |
| hsa-miR-625_st | 47.6 | −3.0 | 0.0072 |
| hsa-miR-105_st | 34.0 | −2.6 | > 0.0001 |
| hsa-let-7i_st | 264.9 | −2.6 | 0.0003 |
| hsa-miR-28-5p_st | 43.7 | −2.4 | 0.0001 |
| hsa-miR-185_st | 312.2 | −2.4 | >0.0001 |
| hsa-miR-16_st | 518.3 | −2.2 | >0.0001 |
| hsa-miR-130b_st | 154.7 | −2.2 | 0.0012 |
| hsa-miR-23a-star_st | 39.0 | −2.2 | 0.0299 |
| hsa-miR-629_st | 39.2 | −2.1 | 0.0027 |
| hsa-miR-1180_st | 28.3 | −2.0 | 0.0007 |
| hsa-miR-106a_st | 580.9 | −1.9 | >0.0001 |
| hsa-miR-181b_st | 72.9 | −1.8 | 0.0350 |
| hsa-miR-17_st | 630.9 | −1.8 | 0.0001 |
| hsa-miR-107_st | 714.0 | −1.7 | 0.0001 |
| hsa-miR-26a_st | 758.2 | −1.7 | >0.0001 |
| hsa-miR-203_st | 194.0 | −1.7 | 0.0006 |

**Table S1.** *Cont.*

| miRNA | Difference of Arbitrary Expression | Fold Change | *p*-value |
| --- | --- | --- | --- |
| hsa-miR-103_st | 777.1 | −1.7 | 0.0001 |
| hsa-miR-500_st | 25.3 | −1.7 | 0.0350 |
| hsa-miR-532-5p_st | 57.8 | −1.6 | 0.0220 |
| hsa-miR-93_st | 296.2 | −1.6 | 0.0027 |
| hsa-miR-24_st | 1979.1 | −1.6 | 0.0001 |
| hsa-miR-151-3p_st | 52.5 | −1.5 | 0.0048 |
| hsa-miR-183_st | 49.4 | −1.5 | 0.0148 |
| hsa-miR-29a_st | 164.8 | −1.4 | 0.0187 |
| hsa-let-7a_st | 813.6 | −1.4 | 0.0043 |
| hsa-miR-25_st | 75.8 | −1.4 | 0.0025 |
| hsa-let-7e_st | 792.9 | −1.3 | 0.0097 |
| hsa-miR-22_st | 91.9 | −1.3 | 0.0101 |
| hsa-miR-23b_st | 465.3 | −1.2 | 0.0214 |
| hsa-miR-23a_st | 741.4 | −1.1 | 0.0217 |
| hsa-miR-99b_st | −383.6 | 1.3 | 0.0005 |
| hsa-miR-423-3p_st | −103.7 | 1.3 | 0.0008 |
| hsa-miR-92a_st | −563.5 | 1.4 | 0.0023 |
| hsa-miR-125b_st | −63.7 | 1.5 | 0.0132 |
| hsa-miR-1275_st | −119.4 | 1.5 | 0.0101 |
| hsa-miR-191_st | −440.2 | 1.5 | 0.0006 |
| hsa-miR-222_st | −579.7 | 1.6 | 0.0003 |
| hsa-miR-342-3p_st | −49.3 | 1.6 | 0.0424 |
| hsa-miR-663_st | −63.1 | 1.7 | 0.0011 |
| hsa-miR-374a-star_st | −70.8 | 1.7 | 0.0029 |
| hsa-miR-197_st | −108.4 | 1.7 | 0.0018 |
| hsa-miR-877_st | −40.5 | 1.8 | 0.0071 |
| hsa-miR-423-5p_st | −62.2 | 1.8 | 0.0039 |
| hsa-miR-487b_st | −87.0 | 1.8 | 0.0017 |
| hsa-miR-320d_st | −148.0 | 1.8 | 0.0012 |
| hsa-miR-768-5p_st | −172.8 | 1.8 | 0.0219 |
| hsa-miR-320a_st | −455.3 | 1.8 | 0.0040 |
| hsa-miR-320b_st | −449.2 | 1.9 | 0.0002 |
| hsa-miR-320c_st | −421.8 | 1.9 | 0.0002 |
| hsa-miR-574-3p_st | −134.0 | 1.9 | 0.0005 |
| hsa-miR-181c-star_st | −163.6 | 2.0 | 0.0045 |
| hsa-miR-638_st | −307.9 | 2.0 | 0.0012 |
| hsa-miR-411_st | −66.1 | 2.0 | 0.0039 |
| hsa-miR-923_st | −1408.2 | 2.0 | 0.0009 |
| hsa-miR-1288_st | −33.2 | 2.1 | 0.0418 |
| hsa-miR-1228-star_st | −147.4 | 2.2 | 0.0044 |
| hsa-miR-132-star_st | −229.3 | 2.2 | 0.0012 |
| hsa-miR-181a-star_st | −202.0 | 2.2 | 0.0007 |
| hsa-miR-149-star_st | −321.5 | 2.4 | 0.0044 |
| hsa-miR-132_st | −329.7 | 2.5 | >0.0001 |
| hsa-miR-768-3p_st | −237.8 | 2.5 | 0.0007 |
| hsa-miR-1246_st | −632.7 | 4.6 | 0.0018 |
| hsa-miR-1290_st | −32.1 | 7.0 | 0.0057 |

**Table S2.** Microarray data from 5 day lysate treated MKN74 cells. Only the differentially expressed miRNAs filtered by difference of arbitrary expression ≥20 and *p*-value ≤0.05
are shown.

| miRNA | Difference of Arbitrary Expression | Fold Change | *p*-value |
| --- | --- | --- | --- |
| hsa-miR-320c_st | 114 | −1.3 | 0.0451 |
| hsa-miR-151-3p_st | 31 | −1.3 | 0.0476 |
